# The effect of retrosplenial cortex lesions in rats on incidental and active spatial learning

**DOI:** 10.3389/fnbeh.2015.00011

**Published:** 2015-02-06

**Authors:** A. J. D. Nelson, E. L. Hindley, J. M. Pearce, S. D. Vann, J. P. Aggleton

**Affiliations:** School of Psychology, Cardiff UniversityCardiff, UK

**Keywords:** retrosplenial cortex, scene learning, navigation, cingulate cortex, visuospatial memory

## Abstract

The study examined the importance of the retrosplenial cortex for the incidental learning of the spatial arrangement of distinctive features within a scene. In a modified Morris water-maze, rats spontaneously learnt the location of an escape platform prior to swimming to that location. For this, rats were repeatedly placed on a submerged platform in one corner of either a rectangular (Experiment 1) or square (Experiments 2, 3) pool with walls of different appearance. The rats were then released in the center of the pool for their first test trial. In Experiment 1, the correct corner and its diagonally opposite partner (also correct) were specified by the geometric properties of the pool. Rats with retrosplenial lesions took longer to first reach a correct corner, subsequently showing an attenuated preference for the correct corners. A reduced preference for the correct corner was also found in Experiment 2, when platform location was determined by the juxtaposition of highly salient visual cues (black vs. white walls). In Experiment 3, less salient visual cues (striped vs. white walls) led to a robust lesion impairment, as the retrosplenial lesioned rats showed no preference for the correct corner. When subsequently trained actively to swim to the correct corner over successive trials, retrosplenial lesions spared performance on all three discriminations. The findings not only reveal the importance of the retrosplenial cortex for processing various classes of visuospatial information but also highlight a broader role in the incidental learning of the features of a spatial array, consistent with the translation of scene information.

Consistent with its interconnectivity with both the hippocampus and the anterior thalamus, the retrosplenial cortex (areas 29, 30) has been repeatedly implicated in spatial learning and memory (Maguire, 2001; Vann et al., 2009). One aspect of spatial memory that has received particular attention with regard to the retrosplenial cortex is scene memory. Functional imaging studies have revealed retrosplenial activity in participants viewing scenes or navigating within virtual-reality environments (e.g., Bar, 2004; Spiers and Maguire, 2006; Epstein et al., 2007; Henderson et al., 2007, 2008; Epstein, 2008). Retrosplenial pathology, particularly in the right hemisphere, is associated with topographic disorientation that manifests as a failure to use landmarks to navigate or orient in both novel and familiar environments, despite preserved familiarity for scenes (Aguirre and D’Esposito, 1999; Maguire, 2001). Whether the retrosplenial cortex is important for compiling scene information or using that information to navigate within the environment remains, however, uncertain. Moreover, the extent to which definitive conclusions can be drawn from neuropsychological studies is constrained by the difficulty of finding cases with circumscribed retrosplenial damage (Vann et al., 2009). These considerations highlight the merit of animal models.

An inherent problem with many rodent spatial memory tasks is that they fail to dissociate between “getting there” and “knowing where” in that the animal is required to navigate to a goal during learning, so that responses made prior to reaching the goal are reinforced. As a consequence, animals may not learn about the spatial relationships between the goal and the surrounding cues in the environment, but rather on subsequent trials animals may simply navigate to the goal by making a sequence of responses elicited by a limited number of cues (Bannerman et al., 1996; Cain et al., 2006; Horne et al., 2012; Gilroy and Pearce, 2014; Kosaki et al., 2014). For example, in the water-maze rats may use the relative distance or angle to a select landmark to reach the goal (Pearce et al., 1998; Hamilton et al., 2007), a strategy potentially available to rats with retrosplenial cortex lesions (RSC; Zheng et al., 2003).

One way to address these problems for water-maze tasks is to train the rats so that the arrangement of available spatial cues is acquired incidentally. In this way, the animal learns the location of an escape platform by being repeatedly placed on the platform without being allowed to swim to the escape platform (Horne et al., 2012; Gilroy and Pearce, 2014; Kosaki et al., 2014). After this initial “passive” training, the rats are allowed to swim to the escape platform for the first time (probe trial). As the rats have no experience of swimming in the water-maze prior to this test trial, their ability to navigate to the escape platform must reflect the development of a spatial memory for the correct location (Horne et al., 2012; Dumont et al., 2014; Gilroy and Pearce, 2014; Kosaki et al., 2014; see also Gaskin and White, 2007). If the retrosplenial cortex is required for creating “mental snapshots” of the spatial elements used to compile a visual scene, then lesion animals should not only struggle to find the correct location, they should also fail to recognize the correct location once there. If, on the other hand, the retrosplenial cortex is important for “getting there”, the lesion animals may have difficulty reaching the correct location but, once there, they should recognize the correct location and show a normal preference for that location. In the probe trial the cues are viewed from a novel perspective (i.e., the center of the pool), thus the probe also taxes the ability to change spatial frames of reference. This ability to translate and change spatial frames of reference has previously been linked to the retrosplenial cortex (Burgess et al., 2001; Byrne et al., 2007; Vann et al., 2009).

The present set of experiments had two principal goals. First, to help distinguish “getting there” and “knowing where”, the experiments assessed the impact of retrosplenial cortex damage on incidental water-maze tasks. The second goal was to assess the importance of the retrosplenial cortex for two quite distinct categories of spatial information, geometric cues or patterned cues.

In Experiment 1, rats were passively trained in a plain rectangular pool to assess the ability to learn geometric properties. The ability to detect geometric relationships, which may underpin the creation of accurate representations of the shape of the environment, is known to depend on the hippocampus and anterior thalamus (O’Keefe and Burgess, 1996; Pearce et al., 2004; Aggleton et al., 2009; Kosaki et al., 2014), both closely interconnected with the retrosplenial cortex. In the rectangular pool, each pair of opposite corners shares the same arrangement with respect to the adjacent long and short walls that form the corner, e.g., long wall to left of a short wall (see Figure 1; Horne et al., 2012; Dumont et al., 2014). The escape platform was located in one of these pairs of corners during passive acquisition. The rats’ ability to use this geometric information was then assessed in a probe trial in which the rat could swim, for the first time, in the rectangular pool. To assess whether the spatial memory used to identify the correct corner in the rectangle depended on local geometric cues, in a subsequent probe the rats were placed in a novel environment (a kite-shaped pool) that contained only some of the same local geometric cues as the rectangle. The rats were next trained “actively” in the rectangle, i.e., allowed to swim to the escape location on every trial. This comparison between the passive and active versions of the task helped test for any gross navigational impairments that could contribute to any observed deficits on the passive probe.

**Figure 1:**
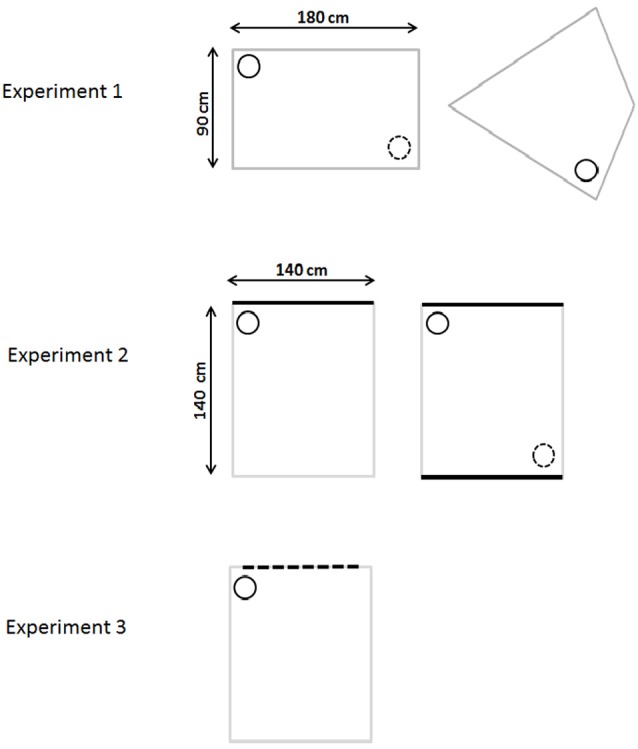
**Schematic diagram of the water-mazes used in Experiments 1–3**. The top row depicts the gray rectangular pool (incidental and active learning) and the kite-shaped pool (for probe trials) used in Experiment 1. Experiment 2 used a square pool with white walls and either one black wall (initial incidental training) or two black walls (active training and probe). Experiment 3 used a square white pool with one wall having vertical black stripes. The small circle represents the location of the platform where the rat was placed during passive training. The small dotted circle represents the other identical (correct) corner (where applicable).

Experiments 2 and 3 focused on the ability to learn the spatial disposition of differently patterned walls in a square pool. Part of the rationale came from the finding of retrosplenial activation in humans when viewing permanent landmarks (Auger et al., 2012). For these experiments the location of the correct corner was determined by the particular juxtaposition of distinctive colored walls, for example a black wall to the right of a white wall (Figure 1; Gilroy and Pearce, 2014). In Experiments 2 and 3 the square maze contained three identical white walls, which contrasted with one black (Experiment 2) or one striped (Experiment 3) wall. As a consequence, it was not sufficient to learn just to approach the black (or striped) wall or the white wall in order to find the correct corner. Rather the rat needed to learn the particular spatial relationships between two walls of equal size but different visual appearance, e.g., black wall to the right of the left wall (Experiment 2, see Figure 1). Subsequent probes then examined variants of the square pool (e.g., in Experiment 2, a square pool with two black walls and two white walls). Finally, these probe trials were followed by active training in the square pool with the escape platform in the same location as had been used for passive learning.

## General methods and materials

### Subjects

The three experiments involved separate cohorts of male Lister-Hooded rats (Harlan, Bicester, UK). There were 27 rats in Experiments 1 and 3, and 28 in Experiment 2. At the time of surgery the rats weighed 290-350 g (Experiment 1), 238–361 g (Experiment 2), and 278–387 g (Experiment 3). Animals were housed in pairs under diurnal light conditions (14 h light/10 h dark) and behavioral testing was carried out during the light phase. Both food and water were available *ad libitum* throughout the duration of the study. Prior to surgery, the rats were handled daily for a week and then randomly assigned to one of two surgical groups: retrosplenial cortex lesions (“RSC”, *n* = 16, Experiment 1–3) or surgical sham [“Sham”, *n* = 11 (Experiment 1 and 3) or *n* = 12 (Experiment 2)]. All experiments were carried out in accordance with the UK Animals (Scientific Procedures) Act, 1986 and EU directive 2010/63/EU.

### Surgery

Animals were first treated with an intraperitoneal injection (i.p) of atropine (0.03 ml of a 600 μg/ml solution, Matindale Pharma, Brentwood, UK) and then deeply anesthetized by an i.p injection (60 mg/kg) of 6% sodium pentobarbital (Sigma Chemical Company, Poole, UK; dissolved in sterile saline and alcohol). Thereafter, anesthesia was maintained with isoflurane (0.5%) in O_2_. The rats were placed in a stereotaxic frame (David Kopf Instruments, Tujunga, CA, USA) with the nose-bar set at +5.0. The scalp was incised and retracted to expose the skull. A craniotomy was performed to expose the cortex. The lesions were produced by injecting a solution of 0.09 m N-methyl-D-aspartic acid (NDMA; Sigma) dissolved in phosphate buffer (pH 7.2) via a 1-μl Hamilton syringe (Bonaduz, Switzerland). Infusions at a rate 0.1 μl/min were made bilaterally at seven sites within the RSC. After each injection, the needle was left *in situ* for 5 min.

The anterior-posterior (AP) coordinates were measured in mm from bregma, the medio-lateral (ML) coordinates (in mm) relative to the sagittal sinus, and the dorso-ventral (DV) coordinates (in mm) with reference to dura. The stereotaxic coordinates (Experiments 1 and 2) at each of the seven sites were as follows: (1) −1.8 (AP), ±0.4 (ML), −1.0 (DV); (2) −2.8 (AP), ±0.4 (ML), −1.1 (DV); (3) −4.0 (AP), ±0.4 (ML), −1.1 (DV); (4) −5.3 (AP), ±0.4 (ML), −2.4 (DV); (5) −5.3 (AP), ±0.9 (ML), −1.4 (DV); (6) −5.3 (AP), ±0.9 (ML), −1.8 (DV); (7) −7.5 (AP), ±1.0 (ML), −1.1 (DV). In Experiment 3 the coordinates were as follows: (1) −1.6 (AP), ±0.4(LM), −1.3 (DV); (2) −2.8 (AP), ±0.5 (LM), −1.3 (DV); (3) −4.0 (AP), ±0.5 (LM), 1.3 (DV); (4) −5.3 (AP), ±0.5 (ML), −2.6 (DV); (5) −5.3 (AP), ±0.9 (ML), −1.6 (DV); (6) −6.6 (AP), ±1.0 (ML), −2.0 (DV); (7) −7.5 (AP), ±1.1 (ML), −1.3 (DV).

In Experiment 1, 0.26 μl of NDMA was injected at each site except at the most posterior site (7) where the volume was reduced to 0.1 μl. In Experiment 2, 0.27 μl was injected at the first three rostral sites, 0.29 μl at sites four to six, and finally 0.1 μl was injected at the most posterior site. In Experiment 3, 0.25 μl was injected in the first three sites, 0.26 μl in the next three sites, and 0.1 μl in the final posterior site.

On completion of the surgery, the skin was sutured and Clindamycin anti-biotic power (Pfizer, Walton Oaks, UK) was applied topically. Subcutaneous Metacam (0.03 ml of a 5 mg/ml solution, Buehringer Ingelheim Lid, Bracknell, UK) provided peri-operative analgesia. Animals also received subcutaneous injections of glucose saline (5 ml). The surgical shams received the identical procedure except that the needle was not lowered into the cortex and no NDMA infusions were made. All rats were allowed a minimum of 10 days post-operative recovery.

### Histology

Histological procedures included the staining of coronal sections for Nissl substance. At the end of behavioral testing, the rats were deeply anesthetized with sodium pentobarbital (60 mg/kg, i.p; Euthatal; Merial Animal Health, Harlow, UK) and then transcardially perfused with 0.1 M phosphate-buffered saline (PBS) at room temperature for approximately 2 min (flow rate 35 mL/min), followed by a 4% solution of depolymerized paraformaldehyde in 0.1 pH for approximately 10 min at a flow rate of 35 mL/min. The brains were removed and post-fixed for 4 h in the same fixative and then cyroprotected in 25% sucrose solution (in PBS) overnight. Four adjacent series of coronal sections (40 μm) were cut on a freezing sliding microtome. Three series were collected and stored in cyroprotectant for subsequent processing. One-in-four series was directly mounted onto gelatin-coated slides and, when dry, stained with cresyl violet, a Nissl stain. The sections were then dehydrated through an alcohol series, cleared with xylene, and cover-slipped with the mounting medium DPX. A second series was stained for NeuN, which is a selective marker for neurons that helps visualize the extent of any lesion (Jongen-Rêlo and Feldon, 2002). This second series was collected in PBS. To visualize NeuN, the free-floating sections were rinsed in 0.1 M PBST (PBS with 0.2% Triton X-100) and treated with 0.3% H_2_O_2_ (hydrogen peroxide) in 0.1 M PBST for 3 min to suppress endogenous peroxidase activity. Sections were rinsed four times in 0.1 M PBST for 10 min each time, and then incubated for 48 h at 4°C in the monoclonal anti-NeuN serum (1:5000; Chemican, Temecula, CA, USA) diluted in PBST. After rinsing four times in 0.1 M PBST for a further 10 min each time, sections were incubated for 2 h in the secondary antibody, avidin-biotin-horseradish peroxidase complex (1:200; ABC-Elite, Vector Laboratories, Orton Southgate, Peterborough, UK) in PBST. After four rinses in 0.1 M PBST and two rinses in 0.05 M Tris buffer, sections were left for 1-2 min in a chromagen solution consisting of 0.05% diaminobenzidine (Sigma; Poole, UK), buffer solution and 0.01% H_2_O_2_ (DAB substrate kit; Vector Laboratories). The reaction was monitored visually and stopped by rinsing in cold 0.1 M PBS. The sections were mounted and dried on gelatin-coated slides. Both sets of sections were then dehydrated through an alcohol series, cleared with xylene, and cover-slipped with the mounting medium DPX.

### Data analysis

Latency to the correct corner and swim speeds were analyzed by between-subjects ANOVA (sham vs. lesion group), and where appropriate with a within-subject factor of session. For the probes, the time spent in the correct and incorrect corners are not independent so these data are not suitable for within subject analysis. Instead, independent sample *t*-tests were used to make between subject comparisons of the time spent in each of the corners separately. The alpha level was set at *p* < 0.05.

## Experiment 1: incidental learning with geometric cues

This experiment examined whether rats with lesions in the retrosplenial cortex could learn a location based on the geometric properties of the environment. Training was initially passive in that the rats were not allowed to swim to the location during acquisition. The rats then underwent two probes trials: one in the rectangular pool and a second probe in a novel configuration (kite-shaped pool) that contained the same geometric information (Figure 1). The purpose of the second probe (kite) was to examine whether the spatial memory used to identify the correct corner depended on local cues rather than the global properties of the environment (Pearce et al., 2004). The experiment then assessed whether the rats could locate the platform using geometric information when trained “actively”, i.e., when allowed to navigate to the goal.

### Apparatus

Training for the experiment proper took place in a gray rectangular pool, positioned within a circular water-maze. The circular water-maze (200 cm diameter, 60 cm deep) was made of white fiberglass and mounted 58 cm above the floor. The pool was filled with water (24 ± 1°C) to a depth of 27 cm and made opaque by the addition of nontoxic emulsion (Roehm and Haas, UK Ltd., Dewsbury, UK). The water was changed daily. An escape platform (10 cm diameter, 2 cm below water surface) could be placed in the pool. The pool was in a room measuring 440 × 400 cm. Lighting was provided by four floor-mounted spotlights (500 W) as well as eight 45-W spot lights that were located in the circular ceiling above the pool. A curtain hanging from the ceiling around the pool was closed throughout the experiment to occlude distal cues. Swim paths were tracked with a video camera suspended directly above the pool. Data were collected and analyzed on-line with an HVS image analyzer connected to a computer that used Water-maze Software (Edinburgh, UK). The escape platform, which was made from clear Perspex, was 10 cm in diameter and was mounted on top of a column. The column stood on the floor of the pool and the platform surface was 2.5 cm below the surface of the water. A beacon, used in pre-training, could be attached to the platform, 0.5 cm from its edge. The beacon was a black and white striped plastic rod, 1 cm in diameter and 10 cm high.

After initial pre-training, the rats were trained in a rectangular-shaped pool constructed from two gray, long Perspex boards (180 cm long, 59 cm high, and 2 mm thick) and two gray, short Perspex boards (90 cm long, 59 cm high, and 2 mm thick). Each board was placed vertically in the pool and suspended by bars that extended over the edge of the pool. The rectangular pool could be rotated within the circular pool so that the position of the correct location remained constant with reference only to the shape of the rectangular pool. The platform was located in the rectangular pool with its center 25 cm from the appropriate corner on a line that bisected the corner (see Figure 1 for details).

### Procedure

#### Pre-training

All rats underwent 4 days of pre-training in the circular swimming pool. The pre-training served to encourage the rats to stay on the platform so that during subsequent placement training, when the rats had no experience of swimming in the rectangular pool, they did not step off into the water. The platform was always located at least 25 cm from the edge of the pool, while for each of the four daily trials it was located in a different quadrant of the pool. The rats were released from randomly selected locations at the side of the pool. On the first 2 days of pre-training the beacon was attached to the platform and on the final 2 days it was removed.

#### Placement training in the rectangular pool

Rats underwent four trials a day for 12 days of placement training. For each session they were carried into a room adjacent to the test room in groups of four in an opaque, aluminum traveling box. They remained in this box between trials. For each trial, the rat was carried from the box to the pool and placed on the platform. The rat was allowed to stay on the platform for 30 s, undisturbed, before being removed, dried, and returned to the holding box. The intertrial interval was approximately 5 min. For half the rats in each group the platform was located in a corner where a short wall was to the left of a long wall. There were two corners in the rectangle with these geometric properties (see Figure 1). For two trials a day (order randomly selected), the platform was located in the one of these corners and for the two other trials it was located in the diagonally opposite corner, that is the corner with the identical geometric properties. The other half of the rats were placed on a platform in one of the other two corners (i.e., a short wall to the right of a long wall). So that the correct locations could only be identified with reference to the juxtaposition of the walls, the rectangle was always oriented along a North-South or East-West axis and was randomly rotated 90° between trials with the stipulation that for each set of four trials the platform was in one of the four possible locations only once. If rat a failed to remain on the platform during the 30 s placement and swam in the pool, it was dropped from the study.

#### Probe: rectangle

On day 12, the first three trials were conducted as previously described. The fourth trial consisted of a probe trial in the rectangle in the absence of the platform. Before the start of the probe trial the rectangle was rotated, as it had been between all the preceding trials. Rats were released from the center of the pool and allowed to swim for 60 s. In order to analyze the results of the probe trial, circular search zones (30 cm in diameter) were placed in each of the four corners of the rectangle. The center of the zones was positioned 25 cm from a line that bisected the corner. The latency to reach the correct corner was recorded. The percentage of time spent in the zones in the two correct corners (the two geometrically equivalent corners where animals had been placed during training) was calculated and compared with the time spent in the two remaining (incorrect) corners.

#### Probe: transfer test in a kite

To test whether the rats had learnt to locate the platform on the basis of local cues (i.e., the juxtaposition of a long and a short wall) and not the global properties of the testing environment, the rats were then tested in a reconfigured arena that contained one corner that shared the same geometric properties as the rectangle (see Figure 1). Following the probe trial in the rectangle, the rats underwent 1 day of reminder placement training that consisted of four passive trials in the rectangle as described above. On the next day, the rats again had three standard placement trials in the rectangle before a probe trial in a novel environment. For this probe trial, the rectangle was reconfigured into a kite shape that contained one corner with the same geometrical properties (e.g., the corner where the short wall was to the left of a long wall) as experienced during initial training as well as its mirror image (i.e., a short wall to the right of the long wall, see Figure 1). As previously, the rats were placed in the center of the pool and allowed to swim for 60 s in the absence of the platform. The percentage time spent in the correct corner was compared with the time spent in the incorrect corner (i.e., the mirror-image of the correct corner).

#### Active acquisition in the rectangle

The animals received five sessions of active training in the rectangular pool. There were four trials in each session, and for each trial rats were required to escape from the pool by swimming to one of two submerged platforms that were located in the same diagonally opposite corners as during placement training. To start the trial, the rat was placed in the center of the pool facing the middle of one of the walls. Rats were allowed 60 s to locate a submerged platform. If a rat failed to find a platform within the 60 s limit, the experimenter guided the rat to a platform. Rats remained on the platform for 30 s. Between each trial, the rectangular pool was rotated either clockwise or anticlockwise 90°. The latency to reach the platform was recorded.

On day three, a single probe session was conducted in the rectangular pool. The first three trials took place in the manner previously described, i.e., active training. The platforms were then removed and the rat was released in the center of the pool and allowed to swim for 60 s. On day four, the rats underwent four further standard acquisition trials in the rectangular pool. On day five, a single probe trial was conducted in the kite-shaped pool. As previously, the first three active trials took place in the rectangle. Before the probe trial, the platforms were removed and the rectangle was reconfigured into the kite shape and the rats were placed in the center of the pool and allowed to swim for 60 s in the absence of the platform. For both probe trials, the percentage time spent in the correct corners in the rectangle or corner in the kite was compared with the time spent in the incorrect (mirror-image) corners.

## Experiment 1: results

### Histology

**Figure 2:**
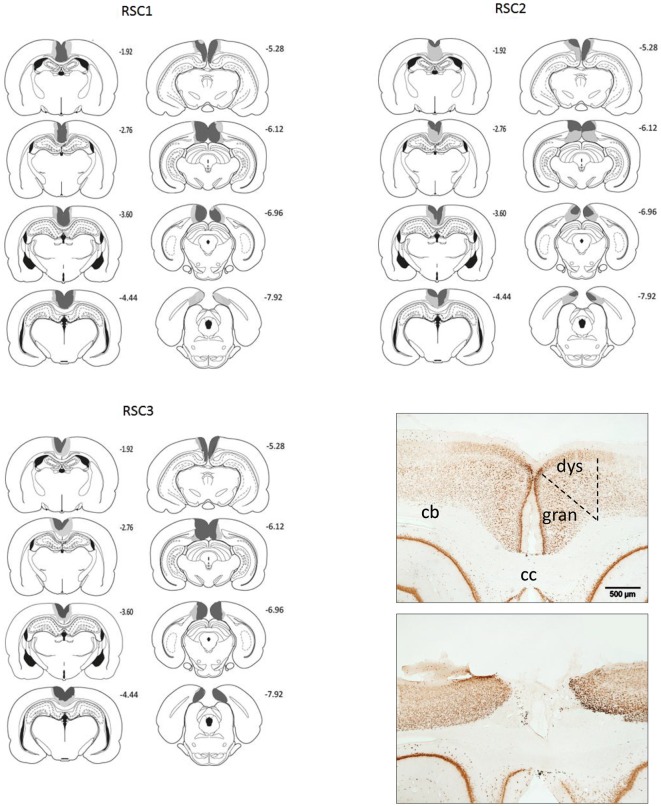
**A series of coronal sections showing those cases with the largest (gray) and smallest (black) lesions in each of the three cohorts (RSC1, RSC2, RSC3)**. The numbers refer to the distance behind bregma in mm. Bottom right: Two photomicrographs of NeuN stained coronal sections. The upper section is from a sham control while the lower case has a mid-sized retrosplenial lesion. The scale bar is 500 μm long. Abbreviations: cb, cingulum bundle; cc, denotes corpus callosum; dys, dysgranular retrosplenial cortex; gran, granular retrosplenial cortex. Figures adapted from Paxinos and Watson (2006).

The borders and the nomenclature for the retrosplenial cortex are taken from Van Groen and Wyss (1990a, 1992, 2003). In Experiment 1 (RSC1, Sham1), a total of three RSC1 rats were excluded from all analyses, as these cases had either excessive sparing within the retrosplenial cortex or extensive bilateral hippocampal and subicular damage. In the remaining 13 cases, the RSC1 surgeries consistently produced marked cell loss throughout almost the entire retrosplenial cortex (Figure 2) and much of the tissue had completely collapsed, but in other areas where tissue was still intact the remaining cells looked abnormal and there was extensive gliosis. Anterior to the splenium, the lesions were essentially complete. Caudal to the splenium, there was partial sparing of granular a retrosplenial cortex in six cases (four bilateral) and in one case there was bilateral dysgranular sparing. Additional cell loss occurred in a discrete part of the most dorsal medial portion of CA1 in the septal hippocampus (six cases bilateral, three cases unilateral). There was also ventricular dilation in five cases.

### Behavior

The salient findings are summarized in Table 1.

**Table 1:** **A summary of the main experimental findings from the passive and active versions of the three tasks used**.

	Passive training		Active training		
	Latency to correct corner	Preference for correct corner	Acquisition	Latency to correct corner	Preference for correct corner
Experiment 1 – rectangle	χ	χ	✔	✔	✔
Experiment 1 – kite	✔	?	–	✔	✔
Experiment 2 – one black wall	✔	χ	–	–	–
Experiment 2 – two black walls	χ	χ	χ	✔	✔
Experiment 3 – striped wall	✔	χ	χ	✔	✔

*✔ denotes no apparent retrosplenial cortex lesion deficit. χ denotes retrosplenial cortex lesion deficit. ? denotes lesion effect close to p < 0.05*.

#### Pre-training

There was no difference in escape latencies between the two groups during the first 2 days of pre-training when the beacon was attached to the submerged platform (*F* < 1). The escape latencies improved from day 1 to day 2 (*F*_(1,22)_ = 37.5, *p* < 0.001) but this improvement did not interact with lesion (*F* < 1). In the absence of the beacon, escape latencies again improved across the 2 days of pre-training (*F*_(1,22)_ = 6.0, *p* < 0.05) but these latencies did not interact with lesion (*F* < 1). There was, however, an overall effect of lesion (*F*_(1,22)_ = 4.5, *p* < 0.05) as the RSC1 group had faster escape latencies relative to the Sham1 group. Mean swim speeds across the 4 days of pre-training did not differ between the two groups (*F* < 1) [mean swim speed cm/s (±S.E.M): RSC1 = 28.7 (±0.6); Sham1 = 28.9 (±0.5)].

#### Rectangle and kite probes

**Figure 3:**
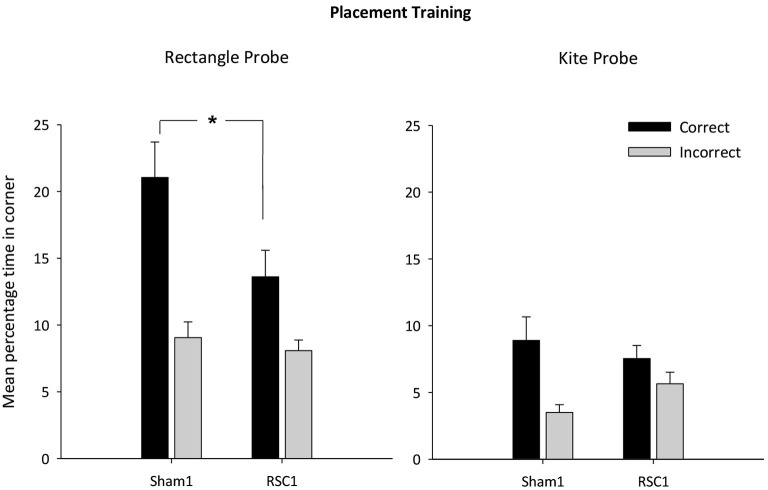
**Experiment 1—Incidental placement training in the rectangular pool**. Probe performance is measured as the mean percentage time (±S.E.M) spent swimming in either the correct or incorrect (mirror-image) corners for both the Sham1 and RSC1 groups during the Probe Tests in the rectangle (left-hand) and kite (right hand). * denotes significant difference between the groups, *t*-test *p* < 0.05.

Initial analysis revealed that there were no differences between the two groups in swimming speeds on either probe (both *F*s < 1). The RSC1 group took longer to first reach the correct corner(s) in the first probe (rectangular pool) relative to the Sham1 group (*F*_(1,22)_ = 5.9, *p* < 0.05) [mean latency s (±S.E.M): RSC1 = 17.3 (±2.0); Sham1 = 10.7 (±1.7)]. For the second probe (kite pool) the latency to first reach the correct corner did not differ by lesion group (*F* < 1) [mean latency s (±S.E.M): RSC1 = 20.8 (±3.9); Sham1 = 15.7 (±4.0)]. In the rectangle (*t*_(22)_ = 2.3, *p* < 0.05) but not the kite (*t*_(22)_ = < 1), sham animals spent longer in the correct corner(s) than the RSC1 animals (Figure 3). Time spent in the incorrect corner did not differ by lesion group in the rectangle (*t* < 1), but in the kite there was a trend towards the RSC1 group spending more time in the incorrect corner relative to the Sham1 group (*t*_(22)_ = 1.9, *p* = 0.059).

#### Active acquisition in the rectangle

**Figure 4:**
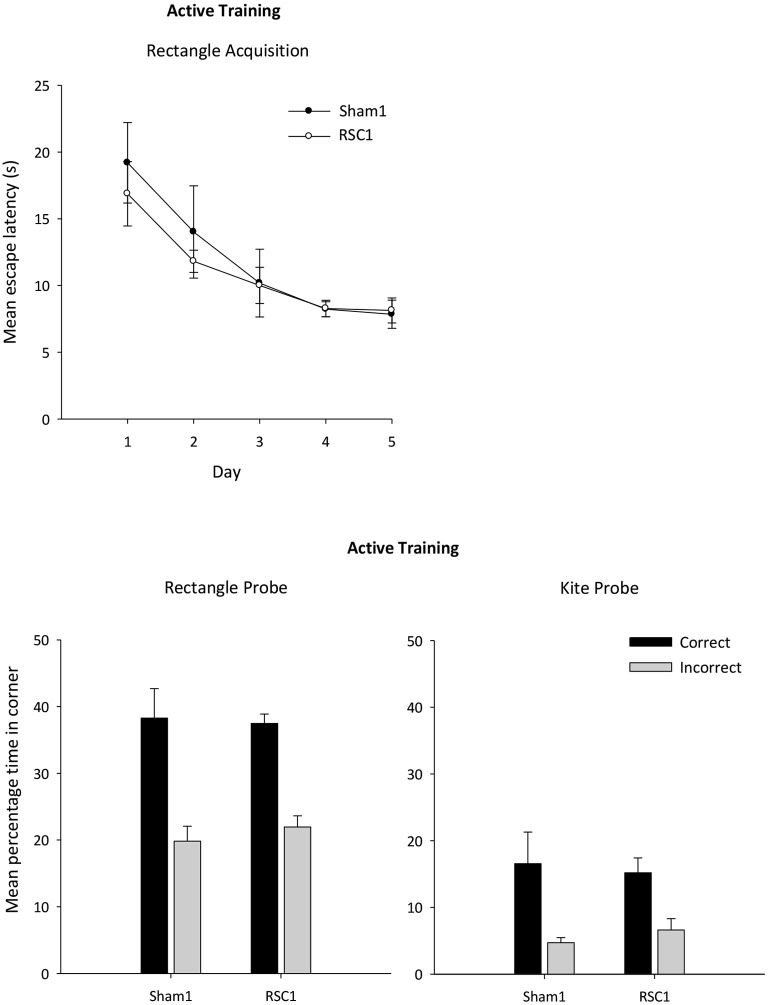
**Experiment 1—Active training in the rectangular pool**. Probe performance is measured as the mean percentage time (±S.E.M) spent swimming in either the correct or incorrect corner(s) (mirror-image) for both Sham1 and RSC1 groups during the Probe Tests in the rectangle (left-hand) and kite (right-hand).

Analysis of the escape latencies across the 5 days of active training in the rectangle (Figure 4) revealed a main effect of day (*F*_(1,88)_ = 14.7, *p* < 0.001) as latencies reduced with training. This improvement was unaffected by lesion group (*F* < 1) and there was no overall effect of lesion (*F* < 1).

#### Active acquisition probes

The mean times spent in the correct and incorrect corner(s) during the probes in the rectangle and kite are presented in Figure 4. There were no group differences in the time in spent in the correct corner(s) in either the rectangle or the kite (*t* < 1). Similarly, time spent in the incorrect corners did not differ by group (*t* < 1).

## Experiment 2. Incidental spatial learning with visual cues: black/white walls

This experiment tested whether rats with retrosplenial cortex damage could passively learn about a spatial location based on the different arrangement of black and white walls (Figure 1). The rats were tested twice: once in a square pool containing a single black wall (one correct corner) and again in a square pool containing two black walls (two correct corners). As with Experiment 1, the purpose of the probe trial in the novel configuration was to assess the extent to which probe performance was driven by a memory of local cues or by the global properties of the testing environment. Lastly, the rats were actively trained in the pool with two black walls.

### Apparatus

The same water-maze and test room was used as in Experiment 1. After initial swim training, the rats were trained in a square-shaped pool constructed from four Perspex boards (each 140 cm long, 59 cm high). One board was painted black and the other three were painted white (Figure 1). Each board was placed in the pool and suspended by bars that extended over the edge of the pool. The square pool could be rotated within the circular pool so that the position of the correct location remained constant with reference to a corner where a black and white wall met. As with Experiment 1, a curtain occluded distal visual cues. The platform was located in the appropriate corner of the square with its center 25 cm from the corner of a line that bisected the corner of the maze (see Figure 1).

#### Pre-training

Pre-training was conducted in exactly the same manner as described for Experiment 1.

#### Placement training in the square pool with black/white walls

Rats received four trials a day for 8 days of placement training in the square pool. The training was identical to Experiment 1 except that the rats were placed on the platform in one corner of the square pool. For half the rats in each group, the platform was located in the corner where the white wall was to the left of the black wall, while for the remaining rats the platform was located in the corner where the white wall was to the right of the black wall.

#### Probe: single black wall

On day 8, the first of the three trials were conducted as standard placement trials. The fourth trial consisted of a probe trial in the square pool in the absence of the platform. The orientation of the square pool was different to the preceding three trials. Rats were released from the center of the pool and allowed to swim for 60 s. In order to analyze the results of the probe trial, circular search zones (30 cm in diameter) were used in each of the four corners of the square. The center of each zone was positioned 25 cm from a line that bisected the corner. The latency to reach the correct corner was recorded. The percentage of time spent in the zone in the correct corner was calculated and compared with the time spent in the remaining (incorrect, i.e., mirror-image) corner.

#### Probe: transfer test square with two black walls

Following the probe in the square pool, the rats underwent 1 day of reminder placement training that consisted of four trials in the square pool with a single black wall as described above. On the next day the rats again had three standard placement trials in the square pool before a probe trial in a novel square pool that contained the same local visual information, i.e., corners where a black and a white wall meet, but now the pool contained two opposing white walls and two opposing black walls (Figure 1). Thus, in the new configuration, there were two corners that shared the same properties as the corner that rats had been placed in during training (e.g., the corner where a white wall was to the left of a black wall, see Figure 1). As with the transfer test in Experiment 1, the aim of this additional probe was to examine whether the spatial memory formed during placement training depended on local cues (i.e., the arrangement of different colored walls) rather than the global properties of the testing arena. As previously, the rats were placed in the center of the pool and allowed to swim for 60 s in the absence of the platforms. The percentage time spent in the two correct corners was compared with the time spent in the two incorrect corners.

#### Active acquisition in the square pool

The animals then received five sessions of active training in the square pool (with two black and two white walls). There were four trials in each session, and for each trial rats were required to escape from the pool by swimming to one of the two submerged platforms that were located in the same corners as during placement training (there was one platform in each of the two corners that shared the same structural properties). The rat was placed in the center of the pool facing the middle of one of the walls. Rats were allowed 60 s to locate the submerged platform. If a rat failed to find the platform within the 60 s limit, the experimenter guided the rat to the platform. Rats remained on the platform for 30 s. Between each trial, the square pool was rotated either clockwise or anticlockwise 90°. The latency to reach the platform was recorded.

On day five, a single probe session was conducted in the square pool. The first three trials took place in the manner previously described. The platforms were then removed and the rat was released in the center of the pool and allowed to swim for 60 s. As in previous probes, the percentage time spent in the four corners (two correct and two incorrect) was calculated.

## Experiment 2: results

### Histology

In Experiment 2, the RSC2 surgeries (Figure 2) produced marked cell loss and extensive gliosis throughout almost the entire retrosplenial cortex. Anterior to the splenium, the lesions were largely complete, except in two cases where there was some granular cortex sparing (one bilateral). Caudal to the splenium there was partial sparing of granular a retrosplenial cortex in five cases (three bilateral). Additional cell loss occurred in a discrete part of the most dorsal portion of CA1 in the septal hippocampus (one case bilateral, five unilateral). In nine cases (three bilateral), narrowing of the medial blade of the dentate gyrus occurred but was confined to the level of the splenium. These same cases showed very restricted cell loss in the dorsal subiculum at the same level. In one animal there was some bilateral thinning of the parietal cortex. No animal was removed on the basis of their surgery, leaving 16 RSC2 and 12 Sham2 animals.

### Behavior

The salient findings are summarized in Table 1.

#### Pre-training

Neither group differed in their time to find the platform during the 2 days of pre-training with the beacon attached to the submerged platform. The rats’ escape latencies improved across the 2 days (*F*_(1,26)_ = 74.2, *p* < 0.001), but there was no interaction with lesion nor an effect of lesion (both *F* < 1). There was also an improvement in escape latencies across the two subsequent pre-training days in the absence of the beacon (*F*_(1,26)_ = 6.2, *p* < 0.05), but again performance was equivalent in the two groups (max *F*_(1,26)_ = 2.6, *p* = 0.12). Across the 4 days of pre-training, there was no difference in swim speeds between the two groups (*F* < 1) [mean swim speed cm/s (±S.E.M): RSC2 = 23.5 (±0.4); Sham2 = 23.7 (±0.4)].

#### Probes

**Figure 5:**
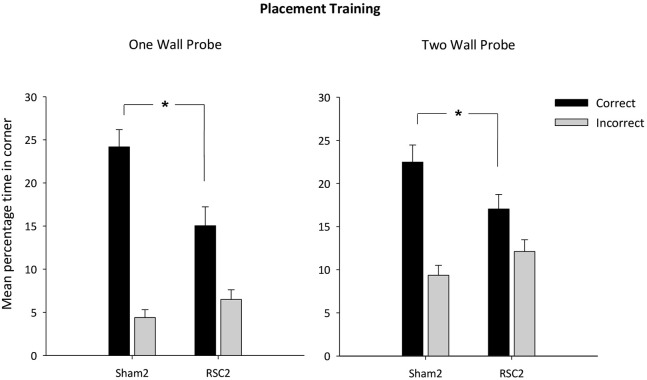
**Experiment 2—Placement training in the square pool with black wall(s)**. Probe performance is measured as the mean percentage time (±S.E.M) spent swimming in either the correct or incorrect (mirror-image) corners for both Sham2 and RSC2 groups during the Probe Tests in the square pool with either one black wall (left-hand) or two black walls (right-hand). * denotes significant difference between the groups, *t*-test *p* < 0.05.

Across the two probes, there was no difference in swim speeds between the two groups (*F*_(1,26)_ = 1.3, *p* = 0.28). The latency to reach the correct corner was not affected by the retrosplenial lesions in the One Wall probe (*F* < 1) [mean latency s (±S.E.M): RSC2 = 16.4 (±2.2); Sham2 = 14.1 (±3.5)], but in the Two Wall probe the RSC2 group took longer to first reach one of the correct corners (*F*_(1,26)_ = 7.9, *p* < 0.01) [Mean latency s (±S.E.M): RSC2 = 19.7 (±2.3); Sham = 10.8 (±1.9)]. The mean times then spent in the correct and incorrect corner(s) during the probe with the single wall and the transfer test in the pool with two black walls are presented in Figure 5. The Sham2 group spent considerably longer in the correct corner(s) relative to the RSC2 group in both the One Wall (*t*_(26)_ = 2.9, *p* < 0.01) and Two Wall (*t*_(26)_ = 2.1, *p* < 0.05) probes, but time spent in the incorrect corner(s) did not differ by group (*t* < 1).

Finally, the One Wall probe also contained two white/white incorrect corners (Figure 1). The two groups did not differ in their times spent in these two incorrect corners (*t*_(1,26)_ = 1.4, *p* = 0.17). Furthermore, the mean total percentage time spent in these white corners (±S.E.M) was RSC2 = 3.34 (±0.6) and Sham2 = 2.1 (±0.6), highlighting how both groups had learnt to not search in the white/white corners.

#### Active acquisition with two black walls

**Figure 6:**
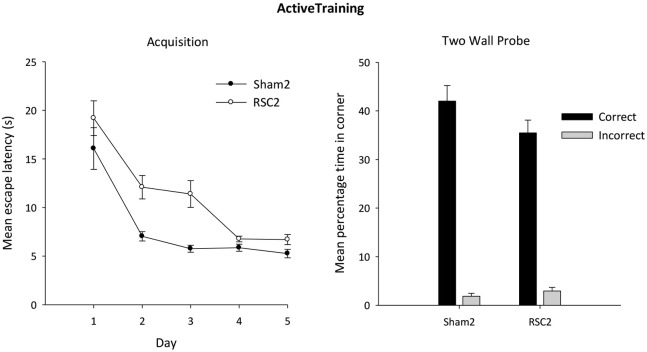
**Experiment 2—Active training in the square pool with two black and two white walls (left side)**. Acquisition performance is measured by the mean escape latencies (±S.E.M) over successive days of training (left-hand). Probe performance is measured as the mean percentage time (±S.E.M) spent swimming in either the correct or incorrect (mirror-image) corners for both the Sham2 and RSC2 groups during the Probe Test in the square pool with two black walls and two white walls (right side).

The RSC2 lesion group initially took longer to find the platform compared to the Sham2 group, but this difference decreased across the 5 days (Figure 6). ANOVA yielded an effect of day (*F*_(4,104)_ = 39.3, *p* < 0.001) and lesion (*F*_(1,26)_ = 12.9, *p* < 0.001) but no day by lesion interaction (*F*_(4,104)_ = 1.9, *p* = 0.11).

#### Active acquisition probe

As is clear from Figure 6, both groups showed a strong preference for the correct corners over the incorrect corners during the probe after active training. Although there is an impression (Figure 6) that the Sham2 performance was superior, this group difference was not confirmed statistically as there was no difference between the groups in time spent in either the correct (*t*_(26)_ = 1.6, *p* = 0.12) or incorrect (*t*_(26)_ = 1.1, *p* = 0.3) corners.

## Experiment 3. Incidental spatial learning with visual cues: striped walls

Like the previous experiment, this experiment also examined the effect of retrosplenial damage on rats’ ability to use visual information (acquired passively) to find a location in a square pool. Rather than containing a single black wall, the pool now had a single black and white striped wall. The appearance of the single wall was changed from Experiment 2 in order to counter the unconditioned preference for dark corners found in previous studies (Dumont et al., 2014; Gilroy and Pearce, 2014).

### Apparatus

Rats underwent pre-training in an identical water-maze to that used for pre-training in Experiments 1 and 2, albeit in a different testing room. For pre-training, the water-maze was located in the center of the test room (300 cm wide × 360 cm long × 240 cm high). All other features of the apparatus and test room were identical to Experiments 1 and 2. Once pre-training had been completed, all subsequent placement training and probe trials occurred in the same room and with the same maze as described for Experiments 1 and 2.

After initial swim training, the rats were trained in a square-shaped pool constructed from four Perspex boards (140 cm long, 59 cm high). Three of these boards were white while the fourth board had black and white vertical stripes. The stripes were made with matt black Fablon (DCFix, UK) and each black stripe was 10 cm wide, with a 10 cm gap between each. At both ends of the board was a white stripe 5 cm wide, i.e., all the corners of the square were white (Figure 1). Each board was placed vertically in the pool and suspended by bars that extended over the edge of the pool. The square pool could be rotated within the circular pool so that the position of the correct location remained constant with reference only to a corner where a striped and a white wall met. The platform was located in the appropriate corner of the square with its center 25 cm from the appropriate corner, on a line that bisected the corner (see Figure 1 for details).

### Procedure

#### Pre-training

Pre-training was conducted in exactly the same manner as described for Experiments 1 and 2.

#### Placement training in the square pool with a striped wall

The placement training in the square pool with one striped wall was identical to Experiment 2. For half of the rats, the corner in which the platform was located was made up of a striped wall on the left and a white wall on the right (from the rat’s perspective), while for the other half of the group the platform was found in the corner made up of a striped wall on the right and a white wall on the left (see Figure 1).

#### Probe: single striped wall

On the eighth day of training, the first three trials were conducted as described above. On the fourth trial the platform was removed from the pool. As previously described, the rats were allowed to swim in the pool for 60 s before being removed and the percentage time spent in the correct and incorrect (i.e., mirror-image) corners of the pool was recorded.

#### Active acquisition: one striped wall

The animals received four sessions of active training in the square pool (one striped wall). This proceeded in exactly the same manner as described for the active training in Experiment 2, with the exception that there was only one striped wall.

On day four, a single probe session was conducted in the square pool with one striped wall. As previously, the first three trials were standard trials in which the rats swam in the pool to locate the submerged platform. The platform was then removed. The rat was then released in the center of the pool and allowed to swim for 60 s. As in previous probes, the percentage times spent in the mirror-imaged corners were calculated.

## Experiment 3: results

### Histology

In Experiment 3, three rats were excluded due to sparing of the retrosplenial cortex or due to extensive bilateral damage to the hippocampus, leaving thirteen RSC3 rats and eleven corresponding Sham3 rats. In the lesion group, extensive cell loss and gliosis was seen throughout the retrosplenial cortex, in both the granular and dysgranular sub-regions. Three RSC3 animals had restricted damage or gliosis in the most dorsal medial tip of the CA1 subfield of the hippocampus (two unilateral). In the remaining case the bilateral hippocampal cell loss was very restricted. Seven animals, including the three with CA1 damage, had slight unilateral thinning of the medial blade of the dentate gyrus just caudal to the splenium. Nine animals had partial sparing of Rga, particularly at its caudal limit. Four rats also had some limited sparing of Rgb. One rat had slight damage to the anterior cingulate cortex at the junction with retrosplenial cortex, while two showed limited unilateral damage to the secondary motor cortex, lateral to the retrosplenial cortex.

### Behavior

The salient findings are summarized in Table 1.

#### Pre-training

Over the course of pre-training, the rats became faster at locating the platform as revealed by a main effect of day (*F*_(3,69)_ = 10.47, *p* < 0.001). There was no main effect of lesion or day by lesion interaction (both *F* < 1), reflecting the equivalent improvement in both lesion and sham animals. Mean swim speeds across the 4 days of pre-training did not differ by lesion group (*F* < 1) [mean swim speed cm/s (±S.E.M): RSC3 = 34.8 (±1.1); Sham3 = 34.5 (±0.9)].

#### Probe

**Figure 7:**
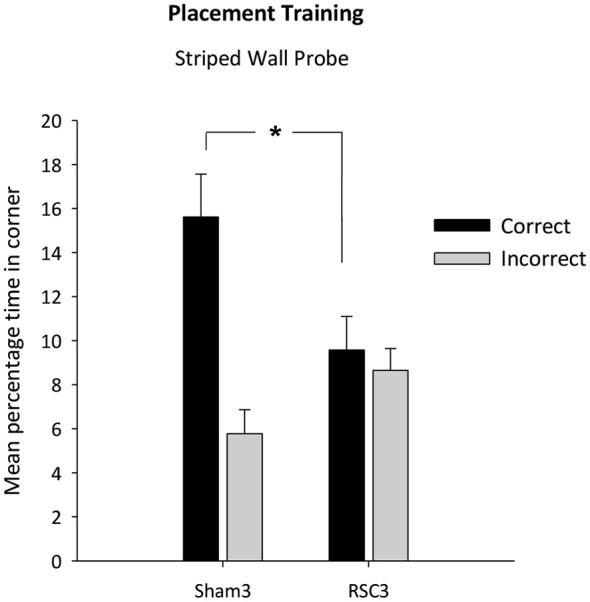
**Experiment 3—Placement training in the square pool with one striped wall and three white walls**. Probe performance is measured as the mean percentage time (±S.E.M) spent swimming in either the correct or incorrect (mirror-image) corners for both Sham3 and RSC3 groups during the Probe Tests in the square pool with one striped wall. * denotes significant difference between the groups, *t*-test *p* < 0.05.

There was no difference in swim speeds between the two groups (*F* < 1). The two groups did not differ in their latencies to first reach the correct corner (*F*_(1,22)_ = 1.78, *p* = 0.19) [Mean latency s (±S.E.M): RSC = 23.6 (±4.2); Sham = 15.3 (±4.6)]. However, as is clear from Figure 7, the Sham3 group spent longer in the correct corner relative to the RSC3 group (*t*_(22)_ = 2.5, *p* < 0.05) and there was a trend towards the lesion group spending longer in the incorrect corner (*t*_(22)_ = 1.9, *p* = 0.063).

In contrast, the two groups did not differ in terms of the time spent in the white/white corners (*t*_(22)_ = 1.2, *p* = 0.25) [mean total percentage time spent in the white corners (±S.E.M): RSC2 = 2.76 (±0.8); Sham2 = 4.1 (±0.8)].

#### Active acquisition

**Figure 8:**
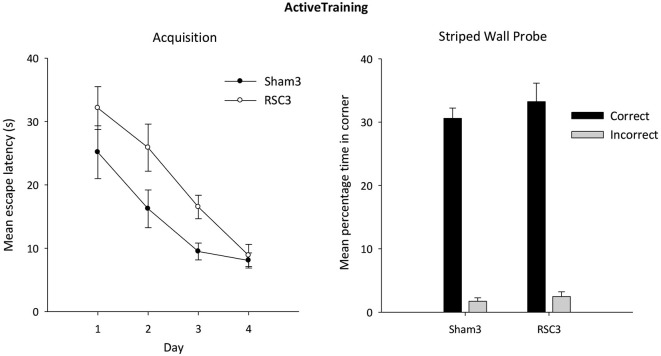
**Experiment 3—Active training in the square pool with one striped wall and three white walls (left side)**. Acquisition performance is measured by the mean escape latencies (±S.E.M) over successive days of training (left-hand). Probe performance measured as mean percentage time (±S.E.M) spent swimming in either the correct or incorrect (mirror-image) corners for both Sham3 and RSC3 groups during the Probe Test in the square pool with one striped wall and three white walls (right side).

Analysis of the escape latencies across the 4 days of active training in the square pool with striped walls revealed a main effect of day (*F*_(3,66)_ = 27.3, *p* < 0.001; Figure 8). Both groups improved across training days and there was no day by lesion interaction (*F* < 1). Nevertheless, the RSC3 group took longer to locate the platform as there was a main effect of lesion (*F*_(1,22)_ = 5.1, p < 0.05) but by the final day of training there was no difference between the two groups (*F* < 1).

#### Active acquisition probe

In the subsequent probe, both groups showed a strong preference for the correct corner (Figure 8). There was no difference between the groups in terms of time spent in either the correct (*t* < 1) or incorrect (*t* < 1) corner.

## Discussion

The current experiments examined the impact of RSC lesions on the ability of rats to learn the location of a spatial goal with reference to either the geometric properties of the surrounding environment (rectangular pool, Experiment 1) or the relative positions of different walls with distinctive appearances (square pool, Experiments 2, 3). In standard water pool problems, rats are presumed to navigate to the submerged escape platform by learning the relationships between multiple, distal spatial cues that fix the location of the platform (Morris, 1981), but this interpretation has been questioned (Hamilton et al., 2007). Unlike standard water pool learning, the present rats were trained “passively” as they were repeatedly placed on the submerged escape platform in a stimulus controlled environment without being allowed to swim to that location (see also Horne et al., 2012; Gilroy and Pearce, 2014). As the rats had no prior experience of navigating in the pool, test performance presumably depends on the spatial properties of the platform location. The aim was, therefore, to preclude, as much as possible, contributions from simple stimulus response learning (Horne et al., 2012; Gilroy and Pearce, 2014; Kosaki et al., 2014), as well as procedural learning (Eichenbaum et al., 1990; Bannerman et al., 1996; Cain et al., 2006), and so better isolate selected aspects of incidental spatial learning.

In Experiment 1, rats were trained passively to discriminate between the corners of a rectangular pool. Although the retrosplenial lesion group showed a preference for the correct location, these rats took longer to reach the correct corners when first allowed to swim in the pool. Furthermore, their preference for the correct corners was attenuated relative to that of the sham group. To help appreciate whether the rats relied on global or local geometric features, all rats were then given a transfer test in a kite-shaped pool. This novel pool contained just one corner that shared the same geometric properties as the correct corner in the original rectangle (e.g., where the short wall is to the left of the long wall), as well as one mirror-imaged incorrect corner. A preference for the correct corner in the kite pool, as shown by the sham rats, suggests that the initial placement training resulted in the use of local geometric cues (see also Horne et al., 2012).

Next, the rats were trained actively in the rectangular pool to find the correct corners, i.e., repeatedly swam until they found the platform. Despite initial deficits reflecting transfer effects, the RSC lesions did not appear to affect subsequent performance. This apparent dissociation between passive (impaired) and active (unimpaired) learning (Table 1) may reflect the different ways that the tasks can be acquired. Unlike the passive tasks in Experiment 1, the active versions can be solved by swimming to a long wall and turning left, for example (see Figure 1; Pearce et al., 2004). While this particular solution involves length discriminations it does not demand that the precise spatial relationships within the maze, e.g., long wall to the left of short wall, be learnt. Given this difference, it is informative that hippocampal and anterior thalamic lesions can impair both the passive and active learning versions of the geometric (rectangular pool) task (McGregor et al., 2004; Pearce et al., 2004; Jones et al., 2007; Aggleton et al., 2009; Dumont et al., 2014; Kosaki et al., 2014).

It has been suggested that an inability to discriminate relative lengths may underlie these hippocampal and anterior thalamic lesion deficits in the rectangular pool (Aggleton et al., 2009; Dumont et al., 2014; Kosaki et al., 2014). If this explanation is correct, it leaves the question of why the retrosplenial cortex, which is densely interconnected to both the hippocampus and anterior thalamic nuclei (Van Groen and Wyss, 1990a,b, 1992, 2003; Wyss and Van Groen, 1992), is not also necessary for this function. While the ability of head direction cells to distinguish different shaped environments (Taube et al., 1990) may partly explain the outcome of anterior thalamic and hippocampal lesions, it has been found that rats with lateral mammillary body lesions are only transiently impaired on the active version of the same geometric task in the rectangular pool (Vann, 2011). This target site is relevant as it propagates head-direction signals to the anterodorsal thalamic nuclei and, thence, to the hippocampal formation (Goodridge and Taube, 1997; Blair et al., 1999; Taube, 2007), so suggesting only a minor contribution from this system to these geometric problems. While it is the case that head-direction cells are also present in retrosplenial cortex (Chen et al., 1994; Cho and Sharp, 2001), the parallel pathways downstream from the anterodorsal nucleus to the hippocampal formation (Domesick, 1970; Shibata, 1993; Taube, 2007) may help explain the lack of any apparent retrosplenial lesion effect on the active version of this task. These same pathways are implicated by the finding that fornix lesions spare active learning in the rectangular pool (Aggleton et al., 2009), so placing emphasis on nonfornical interactions between the anterior thalamic nuclei and hippocampus (Van Groen and Wyss, 1990c; Shibata, 1993; Wright et al., 2010).

Experiments 2 and 3 assessed the spontaneous ability to distinguish locations that differed by whether the contrasting wall (black in Experiment 2, striped in Experiment 3) was to the left or the right of the white walls (Dumont et al., 2014; Gilroy and Pearce, 2014). In both experiments the RSC lesions affected performance on the probe trials after passive training. With the black wall, the retrosplenial rats showed a preference for the correct corner but this preference was significantly attenuated. With the striped wall, the rats with retrosplenial lesions failed to show any preference for the correct corner over its mirror-image. Other lesion effects were seen in the delayed latency to first reach the correct corner on the transfer test in Experiment 2 involving two black and two white walls. In both experiments, this lesion–induced difficulty with passive training then carried over into the beginning of active training but disappeared over successive test days and was absent in the subsequent probe tests. Thus, as with the geometric task, there was an apparent dissociation between passive (impaired) and active (unimpaired) performance. Once again, the active versions of these tasks could be solved by response strategies (e.g., swim to a black wall and turn left) that do not necessarily depend on the formation of a spatial scene. Thus, taken together, the results from all three experiments indicate that RSC lesions impair the first use of incidentally acquired spatial information, but spare active learning that may involve simpler response rules.

The retrosplenial lesions appeared to cause the most severe impairment on passive learning in Experiment 3 (striped wall). One possible explanation is that the striped wall is a less salient and more ambiguous visual cue than the black walls used in Experiment 2. The ambiguity stems from the white elements in the striped wall and the white:white edges at all four corners. The retrosplenial cortex, in particular the dysgranular subregion (area 30), receives visual information directly from the geniculostriate and tecto-cortical visual systems (Vogt and Miller, 1983; Van Groen and Wyss, 1992; Wyss and Van Groen, 1992). In line with this connectivity, there is good evidence that the retrosplenial cortex is important for the processing of visual information as consistent deficits often only emerge on spatial tasks when lesioned animals are forced to rely on allocentric cues (e.g., Sutherland et al., 1988; Warburton et al., 1998; Vann and Aggleton, 2002, 2004; Van Groen et al., 2004; Hindley et al., 2014a).

The deficit in Experiment 3 in the passive stage of the task is striking, not only because it was more severe than the mild impairments seen in Experiments 1 and 2 but also because anterior thalamic damage can spare performance of this task (Dumont et al., 2014). This task sensitivity may relate to the finding that tasks which tax the use of overlapping or ambiguous cues in the absence of any navigational requirement are also sensitive to the effects of retrosplenial cortex damage (e.g., Hindley et al., 2014a; Nelson et al., 2014). The suggestion that the striped wall was a less salient or a more ambiguous cue is supported by the results of the probe test in Experiment 2, where latency to reach the correct corner was only disrupted when the animals were tested in the reconfigured square pool with two black walls (having first been passively trained in a square with only one black wall). Presumably, the second black wall increased the ambiguity of the problem, due to the novelty of the cue configuration as well as the addition of two further potential corners in which the platform could be located.

Across all three experiments, the clearest and most consistent deficits were found in the initial probe after passive training i.e., when the animals swam to the escape location for the first time. While the retrosplenial lesion group was often still able to recognize the correct location when required to use either geometric or salient visuo-spatial information (Experiments 1, 2) their discrimination was diminished or, in the case of Experiment 3, absent. Although this pattern of results suggests that the retrosplenial cortex may be required for the incidental learning of scenes composed of ambiguous or low-salient features, the lesion animals’ ability to recognize the correct locations once there (Experiments 1, 2) suggests that the effects of retrosplenial cortex damage is more specific. This suggestion accords with evidence that patients with retrosplenial damage retain a sense of familiarity for both landmarks and scenes (Maguire, 2001). Furthermore, it seems most unlikely that this pattern of results simply reflects a gross navigational impairment as the retrosplenial lesions spared performance in probes following active training (Experiments 1–3). Indeed, RSC lesions can impair other spatial tasks with little or no navigational component (e.g., Vann and Aggleton, 2002; Hindley et al., 2014a) but can also spare performance on tasks with clear navigational demands (e.g., Neave et al., 1994; Zheng et al., 2003; Pothuizen et al., 2008; Elduayen and Save, 2014).

An aspect of the probe tests not yet been considered is that the rats not only need to learn the array of cues associated with the correct location but must also then use this information to navigate to that location from a novel site (the middle of the pool). To perform the latter task, the rats must change spatial frames of reference as the pool wall cues are viewed from a novel perspective. It might be supposed that similar abilities are taxed in the novel pool probes (kite, Experiment 1, two black walls Experiment 2), which were both associated with deficits. The ability to translate and change spatial frames of reference has previously been linked to the retrosplenial cortex (Burgess et al., 2001; Byrne et al., 2007; Vann et al., 2009). Support for this proposal comes from fMRI studies showing that the retrosplenial cortex is activated during tasks requiring people to imagine looking at the same scene from different viewpoints (Epstein et al., 2007; Lambrey et al., 2012) as well as viewing the mirror-reversal of a previously seen scene (Dilks et al., 2011). A study of participants navigating in virtual reality showed retrosplenial cortex activation specifically when topographical representations were updated or manipulated for route planning (Spiers and Maguire, 2006). Patients with retrosplenial damage report an inability to orient themselves in familiar environments or retrieve coherent spatial representations of the environment (Takahashi et al., 1997; Maguire, 2001). Indeed, a broader role for the retrosplenial cortex in translation between different representations of the same event is suggested by findings that the rat dysgranular retrosplenial cortex is required for cross-modal recognition memory (Hindley et al., 2014b). It should be acknowledged that on the basis of the current results it is not entirely clear whether the deficits reported here arise from impoverished encoding of the visuospatial information necessary to navigate at test or an ability to change spatial frames of reference. One way of addressing this critical issue would be to temporarily inactivate the retrosplenial cortex during either the placement training stage or at test. This approach would explicitly address the issue whether the retrosplenial cortex is required to acquire the spatial information necessary to navigate to the correct location, or whether the retrosplenial cortex is selectively engaged at test when the animal has to change spatial frames of reference and translate this representation when starting from a novel position in the environment. Alternatively, the retrosplenial cortex may be important for both processes.

Taken together, the current set of findings implicate the retrosplenial cortex in the incidental learning of the disposition of spatial features within a scene, an aspect of learning intrinsically tied to episodic memory in people (Gaffan, 1994; Burgess et al., 2001; Byrne et al., 2007; Hassabis and Maguire, 2007; Vann et al., 2009).

## Conflict of interest statement

The authors declare that the research was conducted in the absence of any commercial or financial relationships that could be construed as a potential conflict of interest.
